# Peak Inspiratory Flow Capability for Simulated Dry Powder Inhaler Resistances in Asthma Patients Prescribed Pressurized Metered-Dose Inhalers with Valved Holding Chambers: The USE-DPI Study

**DOI:** 10.3390/jcm15083131

**Published:** 2026-04-20

**Authors:** Lara Bravo Quiroga, José Miguel González Moro, Francisco Javier Álvarez-Gutiérrez, Krasimira Baynova, Mariam De La Poza Abad, José Luis Izquierdo Alonso

**Affiliations:** 1 Hospital Universitario Príncipe de Asturias, Alcalá de Henares, 28805 Madrid, Spain; lbquiroga84@gmail.com (L.B.Q.); respirama@yahoo.es (J.M.G.M.); 2 Escuela de Doctorado, Universidad de Alcalá, 28801 Madrid, Spain; 3 Departamento de Medicina y Especialidades, Universidad de Alcalá, 28801 Madrid, Spain; 4 Hospital Universitario Virgen del Rocío, 41013 Sevilla, Spain; fjavieralvarez2008@gmail.com (F.J.Á.-G.); krasi1024@yahoo.com (K.B.); 5 Centro de Salud Doctor Carles Ribas, 08038 Barcelona, Spain; mariamdelapoza@gmail.com; 6 Hospital Universitario de Guadalajara, 19002 Guadalajara, Spain

**Keywords:** asthma management, peak inspiratory flow, dry powder inhaler, valved holding chamber, inhalation technique

## Abstract

**Background**: Inhaled therapy is the mainstay of asthma management, yet many patients are prescribed pressurized metered-dose inhalers (pMDIs) with valved holding chambers (VHCs) based on a presumed low inspiratory capacity, often without objective measurement. The USE-DPI study aimed to determine how many of these patients can generate sufficient peak inspiratory flow (PIF) to use a dry powder inhaler (DPI). **Methods**: This multicenter, observational, cross-sectional study included 346 patients with asthma treated with pMDI and VHC. PIF was measured using the In-Check Dial at two resistance settings (R2 and R4). The primary outcome was the proportion of patients achieving PIF ≥ 30 L/min. **Results**: Almost all patients reached the 30 L/min threshold (99.4% at R2 and 98.7% at R4). Using a higher threshold of 60 L/min (R2), 76.1% met this criterion. Lower PIF (<60 L/min) was associated with older age, reduced lung function (FEV1 ≤ 80% predicted), and poorer asthma control. No significant variables were associated with failure to reach 30 L/min. **Conclusions**: Most patients using pMDI with VHC can generate sufficient inspiratory flow for medium- to high-resistance DPIs. Objective PIF assessment may help guide inhaler selection, although its clinical impact requires further study.

## 1. Introduction

Inhalation therapy is the treatment of choice for chronic respiratory diseases such as asthma, as it allows for direct drug delivery to the target organ using lower doses and resulting in fewer systemic side effects [1]. Nevertheless, between 30% and 90% of patients commit at least one significant error during the inhalation technique, which reduces clinical efficacy and promotes non-adherence [2]. In pressurized metered-dose inhalers (pMDIs), one of the most frequent critical errors is poor coordination between actuation and the onset of inspiration, although other relevant errors include inadequate exhalation before inhalation, insufficiently slow/deep inhalation, and failure to maintain an adequate post-inhalation breath-hold. When a valved holding chamber (VHC) is added, coordination demands are reduced, but cleaning, portability, and daily handling become more complex. In contrast, for dry powder inhalers (DPIs), a major critical error is failure to generate sufficient peak inspiratory flow (PIF) to disperse the powder effectively [2,3,4]. In the systematic review by Sanchis et al., data were collected regarding technique errors for both pMDIs and DPIs in 54,354 patients. Generally, more errors are detected with pMDIs, that are primarily associated with the coordination between device actuation and the onset of inspiration. In contrast, for DPIs, the most frequent critical error is the failure to generate sufficient peak inspiratory flow (PIF) to disperse the powder [2].

In clinical practice, DPIs are often avoided when there are concerns about insufficient inspiratory flow or a patient’s ability to generate adequate inhalation effort. Clinicians may instead favor pMDIs used with VHCs, reflecting both longstanding familiarity and limitations in available DPI formulations. Although VHCs can address some delivery issues, their size and handling requirements may reduce adherence, particularly in ambulatory or community setting [4,5,6]. Furthermore, the exclusion of DPIs not only restricts patient preferences but may also limit therapeutic options, as not all inhaled asthma medications are available in MDI systems. On the other hand, while pressurized metered-dose inhalers (pMDIs) contribute significantly to the carbon footprint of asthma care due to high-global-warming-potential propellants, transitioning to dry powder inhalers (DPIs) can reduce treatment-related equivalent emissions by over 50% to 95% while maintaining or improving clinical outcomes [7].

The performance of a DPI depends on the relationship between patient-generated flow, device resistance, and the turbulence produced within the device itself. A low-resistance device requires high flow, while a high-resistance device requires lower flow [8,9,10]. This is explained by the phenomenon of turbulent aerosolization, which is fundamentally mediated by airflow velocity and device resistance. Consequently, some devices can function optimally with a PIF of 30 L/min, a threshold that most patients with asthma might be able to reach [8,9,11].

In a proof-of-concept study (INSPIRE), we observed that most patients with asthma were able to achieve a PIF ≥ 30 L/min following brief technical instruction [12]. These findings suggest that a significant proportion of patients treated with a pMDI plus VHC could effectively manage a DPI, thereby simplifying their therapeutic regimen. Nevertheless, evidence from real-world clinical practice remains limited.

The USE-DPI study aimed to quantify, in asthma patients currently prescribed pMDI with VHC in routine care, the proportion able to generate adequate PIF for simulated DPI resistance profiles after briefing standardized instruction.

## 2. Material and Methods

This study was designed to evaluate inspiratory-flow capability under standardized testing conditions in asthmatic patients treated in routine practice with a pMDI and a VHC.

### 2.1. Study Population

This was a multicenter, observational, cross-sectional study in a real-world population of asthma patients treated with pMDI and VHC. The study was conducted in outpatient clinics of Pulmonology, Allergy, and Primary Care, involving stable patients diagnosed with bronchial asthma according to the GINA 2022 criteria. A total of 70 centers participated, each enrolling 5 patients over the age of 6 who were receiving any inhaled medication via pMDI and VHC and had undergone spirometry within the previous 12 months. Patients using other devices without a VHC, those with disabling comorbidities, or those with cognitive impairment that, in the investigator’s judgment, compromised participation were excluded.

Reasons for VHC prescription were recorded in the eCRF from the treating clinician’s documented indication in the medical record and, when needed, were complemented during the study visit by investigator confirmation with the patient or caregiver.

Although a pediatric subgroup was prospectively identified, only 41 participants were younger than 18 years, which limited statistical power for reliable inferential subgroup comparisons. Accordingly, the primary analysis was performed in the pooled population, and all age-specific inferences for pediatric population were avoided.

### 2.2. Procedures

Following the verification of selection criteria and the signing of informed consent, demographic, clinical, and functional data were recorded in an electronic Case Report Form (eCRF).

Participants completed the validated Test of Adherence to Inhalers (TAI) and Feeling of Satisfaction with Inhaler (FSI-10) questionnaires, both previously used in asthma/COPD populations [5,6]. Inspiratory maneuver was assessed under brief standardized instruction using the In-Check Dial (Alliance TechMedical. Burleson, TX. USA). PIF was measured with the In-Check Dial using two simulated resistance settings, R2 and R4, chosen to represent medium-low and medium-high resistance profiles rather than any single marketed DPI.

The investigator instructed each patient on the proper DPI inhalation maneuver (complete exhalation, rapid and deep inhalation, and a 5–10 s breath-hold) and provided training for up to two attempts using the In-Check Dial inspiratory flow meter. Once the technique was deemed adequate, PIF was measured at resistance levels R2 or R4 (alternated between patients). If the 30 L/min threshold was not met, a second attempt was permitted, and the best value was recorded. Inspiratory maneuvers were performed starting from Residual Volume (RV) up to Total Lung Capacity (TLC). To assess the effect of incomplete lung emptying on the inspiratory maneuver, the procedure was repeated with the patient starting inspiration from Functional Residual Capacity (FRC) as an exploratory objective.

The primary endpoint was the percentage of patients achieving a PIF 30 L/min at each resistance level. This threshold represents the optimal peak inspiratory flow (PIF) for high-resistance devices and the minimum requirement for low-to-medium resistance inhalers. Given that the target flow rate for low-to-medium resistance devices typically reaches or exceeds 60 L/min, this variable was specifically evaluated using R2 resistance levels. Secondary variables included the influence of age, sex, body mass index (BMI), pathology and severity, lung function, and comorbidities on maneuver success, as well as clinical criteria for VHC prescription, such as patient satisfaction and awareness of alternative devices.

### 2.3. Sample Size and Statistical Analysis

The primary objective was to analyze the percentage of patients capable of completing an adequate inspiratory maneuver with a dry powder inhaler. Given that 30% to 90% of patients commit at least one significant error in inhalation technique, an estimated value of 50% was used for the sample size calculation to ensure maximum variance. Based on this, and assuming a 95% confidence level and 6% precision using the percentage estimation formula for infinite samples, the minimum required sample size was 267 patients. Accounting for a potential 20% loss to follow-up, the total required sample was 334 patients. For logistical reasons and given the multicenter nature of the study (70 centers), the recruitment target was rounded to 5 patients per center, resulting in a pre-established total of 350 patients.

Analyses were performed using Stata 17 (StataCorp, College Station, TX, USA). Proportions were estimated with 95% CIs. McNemar’s test was used for paired proportion analysis, and multivariable logistic regression was employed to identify factors associated with maneuver success. Candidate covariates for multivariable models were restricted to variables prospectively captured with sufficient completeness and distribution in the eCRF. Variables with sparse data or very low event counts were not forced into the final models to avoid unstable estimates. A *p* value less than 0.05 was considered statistically significant.

### 2.4. Ethical Considerations

The protocol was approved by the Clinical Research Ethics Committee of the Hospital Universitario General de Guadalajara—University of Alcalá (Ref CEIm: 2024.02.EO). The study was conducted in accordance with the Declaration of Helsinki and Good Clinical Practice (GCP) guidelines. All participants (or their legal representatives in patients under 18) provided written informed consent prior to any study-specific procedures.

## 3. Results

A total of 346 patients (305 adults and 41 under 18 years of age) from 70 centers were included in the study. Baseline characteristics are detailed in Table 1. The mean age was 50.5 ± 22.8 years, and 67.9% of the participants were female. Although a pediatric cohort was identified using an arbitrary cutoff of 18 years, the sample size was insufficient to perform meaningful comparative analyses. Consequently, the analysis was conducted as a pooled sample, as both groups exhibited similar trends. Notably, 34.1% of the patients were aged ≥ 65 years. Regarding smoking status, 24% were active smokers and 12% were former smokers; therefore, a concomitant COPD (Chronic Obstructive Pulmonary Disease) component cannot be ruled out in these patients.

### 3.1. Indications for and Knowledge of Valved Holding Chamber Use

The reasons for prescribing a pMDI with a VHC are summarized in Table 2. The most frequent indication was to ensure adequate drug delivery, followed by coordination difficulties and suspected insufficient inspiratory capacity—the latter generally without objective confirmation. Patient knowledge regarding the device was poor; 16% were unaware of alternative inhalation options, and 60% did not know the specific reason for their VHC use. Furthermore, 72% of patients believed they could successfully use an inhaler without a chamber.

### 3.2. Adherence and Satisfaction

**Figure 1:**
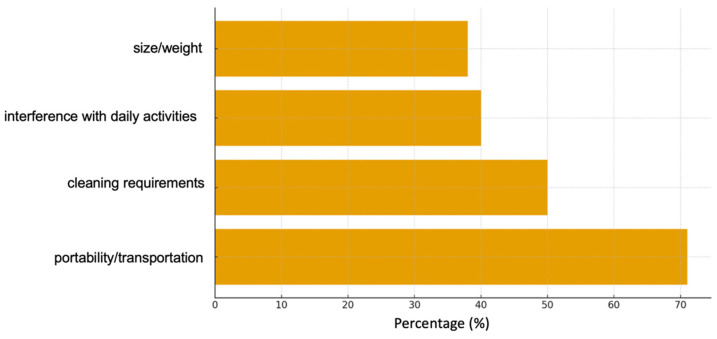
Patient-reported barriers to the use of pressurized metered-dose inhalers with valved holding chambers (categories are not mutually exclusive).

The TAI questionnaire demonstrated good adherence in the majority of patients. Adults exhibited minor fluctuations during asymptomatic periods or while on vacation. Overall satisfaction was high (75%). Figure 1 illustrates the primary limitations perceived by patients, most notably portability/transportation (71%), cleaning requirements (50%), interference with daily activities (40%), and size/weight (38%).

### 3.3. Peak Inspiratory Flow (PIF) Using the Standard Maneuver

**Figure 2:**
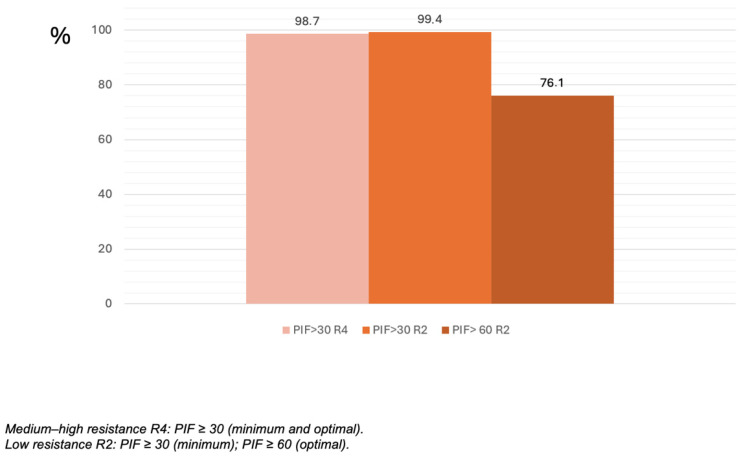
Distribution of Peak Inspiratory Flow (PIF) achieved at R2 and R4 resistance levels following complete exhalation.

Figure 2 shows the distribution of the PIF achieved with R2 and R4 resistances following a complete exhalation. Notably, 98.7% and 99.4% of patients reached a PIF ≥ 30 L/min with the R4 and R2 settings, respectively. The proportion of subjects who failed to reach the ≥60 L/min threshold with R2 resistance was 23.9%. No significant differences were detected between resistance levels regarding the ability to exceed the 30 L/min threshold. However, patients aged ≥ 65 years, those with poorer lung function, or those with worse asthma control exhibited significantly higher proportions of failure to reach the 60 L/min threshold (Table 3).

### 3.4. Impact of Incomplete Lung Emptying

**Figure 3:**
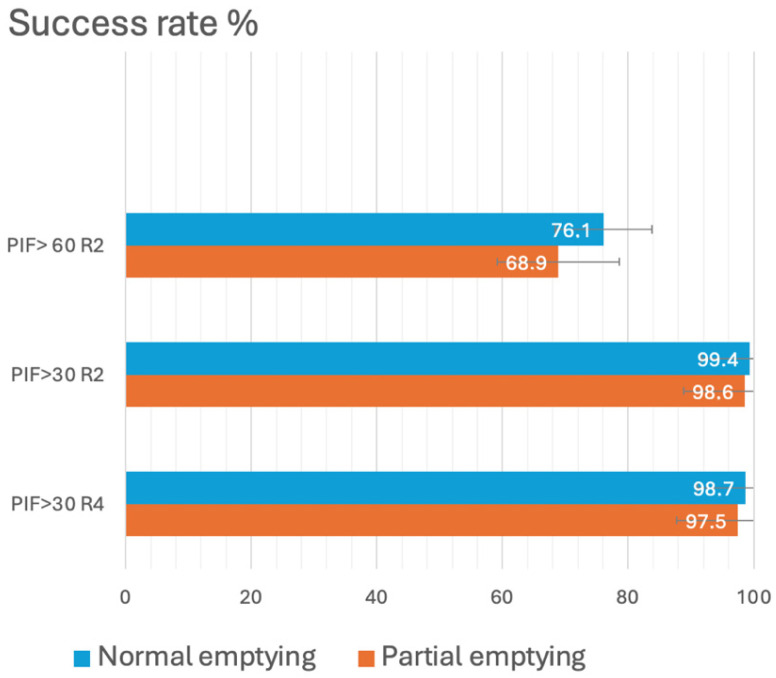
Impact of incomplete lung emptying on Peak Inspiratory Flow (PIF) values: comparison between the standard maneuver and partial exhalation.

The evaluation of incomplete lung emptying showed that, using a PIF threshold of ≥30 L/min, the comparison between the standard maneuver and partial exhalation revealed only a minimal decrease in the success rate (from 99.4% to 98.6% for R2, and from 98.7% to 97.5% for R4). In contrast, when applying a more stringent criterion of ≥60 L/min, 76.1% of patients reached this threshold with the standard maneuver, a figure that fell to 68.9% following partial exhalation (Figure 3).

### 3.5. Analysis of Factors Associated with Maneuver Success

All patients were selected to analyze the factors associated with failure to perform a correct maneuver. With a PIF > 30 L/min as the dependent variable, univariate binary logistic regression models were first performed. For independent variables with *p* < 0.1, a stepwise forward multivariate model was constructed including the following independent variables: age, gender, severity, and control. For the PIF < 30 L/min threshold, no variables were found to be statistically significant or approaching the *p* = 0.05 level in the univariate analysis; therefore, no multivariate analysis was conducted (Table 4a).

Table 4b details the factors associated with a PIF < 60 L/min. Following multivariate analysis, age, lung function and poor asthma control were identified as the only statistically significant independent predictors.

## 4. Discussion

In this multicenter study conducted in a real-world prescribed population, but with standardized inspiratory-maneuver assessment, we observed that most patients with asthma treated with a pMDI and a VHC can generate a PIF sufficient to correctly utilize a high resistance DPI. More than 99% of the sample achieved a PIF ≥ 30 L/min at both medium-low (R2) and medium-high (R4) resistances, confirming that this flow threshold is attainable for nearly all patients following brief, standardized training.

Our findings highlight a significant disconnect between clinical practice and patient functional capacity. Although a clinical suspicion of “insufficient inspiratory capacity” is a primary reason for prescribing a VHC, this perception is rarely supported by objective measurements. In fact, 72% of patients in this study believed they could manage an inhaler without a chamber, and 60% were unaware of the reason why the device had been prescribed. This “prescriptive inertia,” based on subjective perceptions rather than reproducible data, may be unnecessarily limiting therapeutic options and patient autonomy.

The relationship between PIF, the internal resistance of the DPI, and the resulting turbulence is determinant for powder de-aggregation and the fine-particle dose. Previous studies have shown that medium- or high-resistance DPI systems generate adequate aerosolization with relatively modest PIF, whereas low-resistance devices require values exceeding 60 L/min [8,10,13]. This has led to questions regarding the universal application of the 60 L/min threshold, noting that flow requirements depend on device design and resistance [14,15,16,17]. Our findings are consistent with this interpretation: while 30 L/min was a nearly universal threshold, requiring ≥60 L/min significantly reduces the proportion of eligible candidates, particularly among elderly patients and those with poorer lung function. When the 60 L/min threshold was applied, the proportion of successful patients dropped to 76.1% with the standard maneuver, and further to 68.9% following partial exhalation. This pattern mirrors those described in COPD and mixed populations, where 20% to 40% of patients fail to reach this flow with low-resistance devices [13,15,18]. These data reinforce the importance of tailoring DPI selection to individual physiology and considering standardized PIF measurement as an integral part of the prescription process.

A distinctive aspect of this work is the evaluation of the impact of suboptimal technique, specifically incomplete lung emptying prior to inhalation—a frequent occurrence in real-world practice despite theoretical recommendations. Although this maneuver slightly reduces PIF, its impact on the 30 L/min threshold is minimal, decreasing from 99.4% to 97.5%. These findings indicate that inspiratory reserve allows for partial compensation of suboptimal technique when using medium- or high-resistance DPIs. However, the sensitivity of the ≥60 L/min threshold to imperfect exhalation reinforces the risk of under-dosing with low-resistance devices [14,15,16,17,18,19].

Prescription patterns for pMDI + VHC reveal relevant discrepancies: while suspected insufficient inspiratory capacity is frequently cited, it is seldom objectively evaluated. A significant portion of patients are unaware of why they use a chamber, and more than two-thirds believe they could manage without one, aligning with studies describing inhaler selection based more on perceptions than on reproducible metrics [17,18,19]. Adherence and satisfaction were high, consistent with previous data [4,5,6,20,21], although practical limitations related to the chamber (portability, cleaning, inconvenience) were identified as potential barriers to compliance [3,4,5,6,21]. Furthermore, within the current context of commitment to respiratory health and the environment, it is pertinent to note that DPIs lack the hydrofluoroalkane (HFA) propellants found in many pMDIs. Their preferential use significantly contributes to reducing the carbon footprint of asthma treatment, aligning with international sustainability recommendations.

Factors associated with an insufficient PIF to reach 60 L/min were advanced age, more pronounced bronchial obstruction, and poor clinical control, replicating patterns described in both asthma and COPD [15,16,17,18,22]. Recent studies propose PIF as a therapeutic biomarker and suggest that personalizing the inhaler according to PIF improves the device–patient fit [18,22]. From a technological perspective, the literature confirms the robustness of many medium-to-high resistance DPIs against variations in inspiratory flow. In vitro and in vivo studies demonstrate that the emitted dose and fine particle fraction remain relatively stable across a wide range of inspiratory flows [23,24,25,26], supporting the idea that the universal 60 L/min threshold is unnecessarily restrictive for many DPIs.

Additionally, the In-Check Dial, while validated for standardized PIF assessment, does not reproduce all device-specific internal geometries, dispersion mechanisms, or mouthpiece characteristics of commercial DPIs. Only two simulated resistance settings were evaluated; therefore, external validity is stronger for resistance classes than for individual products.

The use of pMDIs represents a significant environmental challenge in respiratory medicine, primarily due to the propellants they contain—hydrofluorocarbons HFC-134a and HFC-227ea—which are potent greenhouse gases with a global warming potential 1300 to 3000 times greater than that of CO_2_. In asthma management, short-acting β_2_-agonists (SABAs) delivered via pMDIs are a major contributor to these emissions, often accounting for up to two-thirds of the total inhaler-related carbon footprint. In this context, transitioning to dry powder inhalers (DPIs) or soft mist inhalers offers a substantial opportunity for decarbonization. For example, maintenance therapy with DPIs can reduce asthma-related CO_2_-equivalent emissions by more than 50% compared with pMDI-based regimens. Moreover, replacing SABA pMDIs with as-needed budesonide/formoterol DPIs has been associated with an estimated 95% reduction in the carbon footprint of asthma management, alongside improved clinical outcomes. Accordingly, while the development of low–global warming potential propellants is ongoing, prioritizing the clinical use of DPIs represents an immediate and effective strategy to reduce the environmental impact of asthma care [7,27,28].

The strengths of our study include its large sample size, multicenter representation, inclusion of both pediatric and adult populations, and the systematic assessment of PIF using resistances comparable to commercial DPIs. Limitations include the cross-sectional design, which precludes evaluating long-term performance stability or the clinical effects of switching to a DPI. In fact, because this was a cross-sectional physiological study, our data do not demonstrate that switching from pMDI with VHC to a DPI improves asthma control, adherence, exacerbation risk, or patient-reported outcomes. Those questions require prospective interventional studies with device-specific follow-up. Additionally, the In-Check Dial, while validated, does not exactly replicate every DPI on the market. The In-Check Dial, while validated for standardized PIF assessment, does not reproduce all device-specific internal geometries, dispersion mechanisms, or mouthpiece characteristics of commercial DPIs. In our study only two simulated resistance settings were evaluated; therefore, external validity is stronger for only two simulated resistance settings were evaluated; therefore, external validity is stronger for resistance classes than for individual products.

The inclusion of both adults and children, and the possibility of asthma-COPD overlap among current or former smokers, introduces clinical heterogeneity. Because the pediatric subgroup was small and smoking-related overlap could not be phenotypically resolved within the study design, the pooled results should be interpreted cautiously in this population.

In summary, our results suggest that the prescription of valved holding chambers due to suspected insufficient inspiratory flow is likely overemphasized in routine practice. Simple and reproducible PIF measurement should be integrated into routine patient assessment, allowing for device selection based on physiological criteria rather than subjective perceptions. Accordingly, the present findings should be interpreted as support for resistance-specific device assessment rather than for indiscriminate substitution of pMDI plus VHC by any DPI. Most patients using pMDI with VHC can generate sufficient inspiratory flow for medium- to high-resistance DPIs. Objective PIF assessment may help guide inhaler selection, although its clinical impact requires further study.

## 5. Conclusions

Although most asthma patients managed with pMDI and VHC can achieve peak inspiratory flows (PIFs) compatible with optimal inspiratory flow for medium-to-high resistance DPIs, following brief instruction, these findings are not universally applicable. Specifically, caution is warranted when considering low-resistance devices, which typically necessitate higher inspiratory flows. Consequently, while objective PIF assessment facilitates the identification of candidates for DPI transition, device selection must remain strictly tailored to the specific resistance profile of the inhaler.

## Figures and Tables

**Table 1:** Baseline Characteristics of the Study Population.

Variable	Total (N = 346)	<18 Years (n = 41)	≥18 Years (n = 305)	
N	346	41	305	
Age (years)	50.5 ± 22.8	11.6 ± 3.4	55.8 ± 18.9	
Age at diagnosis (years)	31.6 ± 21.7	6.7 ± 3.2	35.2 ± 20.9	—
Sex (female), n (%)	235 (67.9)	(43.9)	217 (71.1)	<0.001
Exhaled nitric oxide (FeNO)	25 (13.5/40.5)	32 (22.5/57.5)	25 (12/40)	0.077
**Lung Function**				<0.001
FEV1 ≥ 80% predicted	187 (57.2)	38 (92.7)	149 (52.1)	
FEV1 60–80% predicted	117 (35.8)	3 (7.3)	114 (39.9)	
FEV1 < 60% predicted	23 (7.0)	0	23 (8.0)	
**Asthma Severity (GINA 2019)**				0.001
Intermittent (Step 1)	71 (21.6)	14 (34.1)	57 (19.8)	
Mild (Step 2)	100 (30.4)	16 (39.0)	84 (29.2)	
Moderate (Step 3 or 4)	119 (36.2)	11 (26.8)	108 (37.5)	
Severe (Step 5)	39 (11.9)	0	39 (13.5)	
**In the last 4 weeks**				
Symptoms > 2 times/day (yes)	159 (47.9)	18 (43.9)	141 (48.5)	0.585
Nighttime awakenings (yes)	64 (19.3)	9 (22.0)	55 (18.9)	0.643
Rescue inhaler use > 2 times/week	139 (41.9)	23 (56.1)	116 (39.9)	0.049
Activity limitation due to asthma	155 (47.3)	19 (46.3)	136 (47.4)	0.900
**Asthma Control (GINA 2022)**				0.729
Well-controlled	101 (30.4)	11 (26.8)	90 (30.9)	
Partially controlled (1–2)	143 (43.1)	20 (48.8)	123 (42.3)	
Uncontrolled (3–4)	88 (26.5)	10 (24.4)	78 (26.8)	

**Table 2:** Reasons for prescribing pMDI with a Valved Holding Chamber (VHC).

Clinical Reason	% of Patients
Ensuring adequate drug delivery	43.9%
Coordination difficulties	33.2%
Suspected insufficient inspiratory flow	15.6%
Preference of another healthcare professional	30.9%
Other	7.2%

**Table 3:** Patients achieving PIF > 60 L/min with R2 resistance.

Variable	Criterion	%	Criterion	%	*p*
Age (years)	<65	82	≥65	61.4	<0.001
% predicted FEV1	≥80	89	<80	56.7	<0.001
Therapeutic Step	≤2	83.1	≥3	58.4	<0.001
Asthma Control	Well/partial	84.2	Uncontrolled	48.8	<0.001
VHC indicated due to low capacity	No	85.7	Yes	34.5	<0.001

**Table 4:** (**a**). Factors associated with PIF < 30L/min in the study population. (**b**). Factors associated with PIF < 60/min in the study population.

(**a**)				
**Variable**	**Univariate**		**Multivariate**	
	**OR (IC 95%)**	***p***	**OR (IC 95%)**	***p***
Age (years)	1.030 (0.967/1.098)	0.357	-	-
Age (<65 vs. ≥65 years)	1.420 (−/−)	0.997	-	-
Age at diagnosis (years)	1.017 (0.947/1.092)	0.647	-	-
Sex (Male vs. Female)	0.000 (−/−)	0.995	-	-
Lung Function		0.944	-	-
FEV1 ≥ 80%	1	-		
FEV1 60–80%	1.617 (0.100/26.111)	0.735		
FEV1 < 60%	0.000 (−/−)	0.998		
Lung Function			-	-
FEV1 ≥ 80% vs. <80%	1.348 (0.084/21.737)	0.833		
Asthma Severity		0.996	-	-
Intermittent (1)	1	-		
Mild (2)	1.414 (0.087/22.995)	0.808		
Moderate (3/4)	2.3 × 10^6^ (−/−)	0.996		
Severe (5)	2.3 × 10^6^ (−/−)	0.998		
Asthma Severity			-	-
Intermittent (1) vs. Mild/Moderate/Severe (3/4/5)	3.657 (0.226/59.209)	0.361		
Asthma Control		0.996	-	-
Well controlled	1	-		
Partially controlled	0.000 (−/−)	0.996		
Uncontrolled	1.129 (0.070/18.335)	0.932		
Asthma Control			-	-
Well controlled vs. Partially/Uncontrolled	2.312 (0.143/37.355)	0.555		
(**b**)				
**Variable**	**Univariate**		**Multivariate**	
	**OR (IC 95%)**	***p***	**OR (IC 95%)**	***p***
Age (years)	1.03 (1.02/1.05)	<0.001	1.018 (1.003/1.034)	0.017
Age (<65 vs. ≥65 years)	3.66 (2.16/6.182)	<0.001	-	0.746
Age at diagnosis (years)	1.35 (1.01/1.04)	<0.001	-	0.373
Sex (Male vs. Female)	1.47 (0.85/2.56)	0.170	-	-
Lung Function			-	0.217
FEV1 ≥ 80%	1	-		
FEV1 60–80%	5.20 (2.89/9.34)	<0.001		
FEV1 < 60%	10.28 (4.01/29.34)	<0.001		
Lung Function				
FEV1 ≥ 80% vs. <80%	5.83 (3.31/10.26)	<0.001	3.522 (1.824/6.804)	<0.001
Asthma Severity		<0.001	-	0.285
Intermittent (1)	1	-		
Mild (2)	3.72 (1.34/10.38)	0.007		
Moderate (3/4)	7.85 (2.94/20.97)	<0.001		
Severe (5)	3.41 (1.03/11.27)	0.045		
Asthma Severity				
Intermittent (1) vs. Mild/Moderate/Severe (3/4/5)	5.34 (2.07/13.78)	<0.001	-	0.397
Asthma Control		<0.001		0.002
Well controlled	1	-	1	
Partially controlled	1.66 (0.81/3.40)	0.166	1.153 (0.524/2.537)	0.723
Uncontrolled	4.88 (2.37/10.06)	<0.001	3.299 (1.473/7.390)	0.004
Asthma Control				
Well controlled vs. Partially/Uncontrolled	2.66 (1.39/5.10)	<0.001	-	0.916

OR: Odds Ratio; CI: Confidence Interval. FEV1: Forced Expiratory Volume in 1 s.

## Data Availability

The original contributions presented in this study are included in the article. Further inquiries can be directed to the corresponding author.
